# Examining morbidity and mortality trajectory profiles of hypertension, diabetes and dementia across healthcare systems: an analysis of Catalan and German administrative medical data for the years 2010 to 2019

**DOI:** 10.1186/s12963-026-00473-6

**Published:** 2026-03-29

**Authors:** Anna-Victoria Holtz, Jordi Gumà, Iñaki Permanyer, Gabriele Doblhammer

**Affiliations:** 1 German Center for Neurodegenerative Diseases (DZNE), University of Rostock, Demographic Studies, Ulmenstraße 69, Bonn, 18057 Germany; 2 Institute for Sociology and Demography, University of Rostock, Rostock, Germany; 3 Centre d’Estudis Demogràfics (CED), Barcelona, Spain; 4 Catalan Institution for Research and Advanced Studies (ICREA), Barcelona, Spain

**Keywords:** Harmonization, European health data, Health claims, Morbidity, Sequence analysis, Clustering, Left censoring

## Abstract

**Background:**

Hypertension, diabetes and dementia are prevalent morbidities in ageing populations and share complex relationships as risk factors and comorbidities. Understanding their temporal order is essential to understand health inequalities. Routinely collected medical data offers potential for cross-population comparisons, yet their feasibility for examining morbidity and mortality trajectories across healthcare systems remains underexplored.

**Methods:**

We analyzed administrative medical data from a cohort of more than 1.5 million Catalans and a cohort of 250,000 Germans aged 50 years and above. Data from both cohorts covered the years 2005 to 2019. Efforts were made to harmonize the data from the two healthcare systems. Prevalence was estimated for hypertension, diabetes and dementia. Further analyses focused on individuals born between 1930 and 1954 with at least one of the three morbidities between 2010 and 2019. Morbidity and mortality trajectory profiles were identified using sequence and cluster analysis for large datasets, resulting in 11 distinct profiles per population. Birth cohort and sex-specific profile characteristics were evaluated by multinomial logistic regression.

**Results:**

Age-standardized prevalence of the three morbidities was lower for Catalans (hypertension: 22.4%, CI [22.2–22.5%], diabetes: 8.1% [8.0-8.2%], dementia: 2.9% [2.8–2.9%]) compared to Germans (hypertension: 69.4% [69.0-69.8%]; diabetes: 29.4% [29.2–29.7%], dementia: 8.2% [8.1–8.4%]) at age 55 years and above in 2010. Prevalence differences may largely reflect differences in diagnostic recording and data-generation practices. Among 174,798 Catalans and 121,547 Germans born between 1930 and 1954 with at least one of the three morbidities, Catalans were more likely to be initially free from any of the three morbidities, whereas Germans were more likely to begin with hypertension and experienced a higher proportion of morbidity combinations. Profiles within each population showed differences based on birth cohort and sex.

**Conclusions:**

Profound differences in prevalence as well as in morbidity and mortality trajectories existed between Catalonia and Germany between 2010 and 2019, reflecting more favorable health outcomes in Catalonia. Although administrative medical data yield meaningful insights for each population, comparing results across populations demands careful attention to variations in healthcare systems. To fully realize the potential of a European health data space, efforts are required to further harmonize data.

**Supplementary Information:**

The online version contains supplementary material available at 10.1186/s12963-026-00473-6.

## Background

With increasing life expectancy there is a growing interest in understanding the variability in the onset and progression of morbidity over the life course. Spain and Germany are often cited as examples of belonging to the oldest populations in Europe. In Spain — one of the leading countries in life expectancy at birth (2019: men: 81.1 years; women: 86.7 years) [1] — the Catalan region, in particular, has a life expectancy (men: 81.2 years; women: 86.7 years) that exceeds both the national and European average (men: 78.5 years; women: 84.0 years) [2]. Meanwhile, Germany’s life expectancy (men: 78.6 years; women: 83.4 years) [3] remains close to the European average.

Among the most common morbidities in old age are hypertension, diabetes mellitus and dementias [4–7], which all contribute to cognitive and functional loss. According to the European Health Interview Survey (EHIS) 2019, 28.1% of Spaniards and 37.4% of Germans reported to have hypertension at ages 55–64, while this gap narrows and reverses for the oldest: 50.7% (56.9%) of Spaniards and 53.3% (55.8%) of Germans aged 65 years and above (75 years and above) reported hypertension. Diabetes prevalence at ages 55–64 was comparable between the two countries, with 10.2% for Spanish and 11.1% for German people. At higher ages, the Spanish prevalence of diabetes (age 65+: 20.9%; age 75+: 23.4%) exceeded the German one (age 65+: 18.8%; age 75+: 19.8%). In Catalonia, the estimated dementia prevalence using validated electronic health records was 6.0% for those aged 65 years and above in 2016 [8]. In Germany, a study estimated dementia prevalence based on the total population of statutory insured individuals at the Allgemeine Ortskrankenkasse (AOK) between 2017 and 2022. In 2022, the estimated prevalence was 6.9% for individuals aged 65 years and above [9].

Beyond their individual occurrence, hypertension and diabetes are closely interrelated, with each condition more common among individuals with the other [10]— often described as a “chicken-and-egg” relationship [11]. Both conditions are also well-established risk factors for dementia, particularly when they occur in midlife, accounting for roughly 10% of potentially modifiable dementia risk [12]. Evidence consistently showed that individuals with both diabetes and hypertension, regardless of the order of diagnosis of each disease, have an elevated risk of all-cause dementia [13–15] across sexes and age groups [15]. Sex-specific multimorbidity patterns have been observed, with the highest dementia risk among women with hypertension, diabetes, and coronary heart disease, and among men with hypertension and diabetes alone [16]. Although hypertension followed by diabetes was associated with increased mortality, the elevated dementia risk persisted even in competing-risk analyses [15]. In the two populations under study, higher cardiovascular risk, including high blood pressure and type 2 diabetes, has been linked to increased dementia risk among Catalans [17], where as diabetes has been associated with higher dementia risk and antihypertensive treatment with reduced risk among Germans [18]. Beyond their role as risk factors, hypertension and diabetes are also frequent comorbidities of dementia in old age [12, 16, 19–23], in both Catalonia [24] and Germany [25].

Generally, a better understanding of the order of morbidities is required, indicating a need for a longitudinal approach. A comparative analysis of morbidity and mortality trajectories, both within and between populations, may help to reveal health inequalities [26]. Existing research has primarily examined the clustering of specific morbidities cross-sectionally [5, 27], yet has largely neglected to investigate the sequential ordering of morbidities [27, 28]. This temporal dimension is of particular interest, given that health deterioration follows a progressive course over time. The lack of harmonized longitudinal cross-country studies with timely consistent follow-up periods contributes to this research gap in the European context. One complementary data source that can be utilized is routinely collected administrative medical data. Efforts are being made by the European Commission to implement the European Health Data Space, integrating data on health and healthcare use across the European Union. The aim is to provide a harmonized data space for research, innovation, regulation activities and policy-making [29]. However, little is known about the practicability of process-based data in cross-population longitudinal research. Therefore, the present study aims to compare morbidity and mortality trajectories of hypertension, diabetes, and dementia using administrative medical data from the two structurally distinct healthcare systems of Catalonia and Germany to address the following question: What insights can be derived from the cross-population comparison of morbidity and mortality trajectories using administrative medical data?

To answer this question, we, we first explored the prevalence of hypertension, diabetes and dementia in Catalonia and Germany of the elderly population and determined the number of individuals with at least one of the three morbidities. Second, we investigated morbidity and mortality trajectory profiles by the order of morbidities with the help of sequence and cluster analysis for large datasets and explored whether similar profiles existed in both populations. Furthermore, we examined the characteristics of the morbidity and mortality trajectory profiles according to sex and birth cohort.

## Data and methods

### Data

Longitudinal medical claims data from two distinct healthcare systems were obtained: the universal healthcare systems of Catalonia and Germany.

The HEALIN (Health Inequality) cohort includes more than 1.5 million individuals of all ages in Catalonia, Spain, and is representative of the Catalan population as of January 1, 2005 [30]. Individuals were followed from 2005 until the end of 2021. The dataset contained both primary care and hospital records, including those of institutionalized individuals. All officially registered individuals in Catalonia are eligible for public healthcare and have access to primary care centers and hospitals. Hospital records cover acute illnesses and initial diagnoses, while primary care records track chronic diseases over time. Chronic conditions are recorded at first diagnostic confirmation, regardless of subsequent disease progression. Although private healthcare use is not captured, access to sick leave benefits and subsidized medication for chronic conditions requires the consultation of medical professionals in primary care centers, ensuring substantial coverage within the data [30].

The German cohort consisted of a random sample of 250,000 individuals aged 50 years and above insured by AOK (Allgemeine Ortskrankenkasse), Germany’s largest statutory health insurer. The cohort was drawn in the first quarter of 2004 and followed until the end of 2019. Outpatient and inpatient records were available, including those of institutionalized individuals. The sample distribution by federal states was fairly comparable to the total German population as of the beginning of 2004. Individuals from Eastern German federal states, Bavaria and Baden-Württemberg were overrepresented to a small extent, while individuals from North Rhine-Westphalia were underrepresented [31] (see Additional file 1, Table A1). In Germany, it is mandatory to be health insured. In 2018, about 90% of all individuals were statutory insured and about 10% privately. Among the statutory health insured individuals, more than one third (26.8 million) were covered by AOK in 2019 [32]. Medical doctors are obligated to record a diagnosis whenever a condition is diagnosed during a medical consultation, irrespective of whether the condition was already previously assessed. The diagnosis needs to be recorded in order to prescribe treatment or medication.

Both datasets included information on demographics (birth date and sex), diagnosis history (ICD-10, International Statistical Classification of Diseases and Related Health Problems, 10th edition) from inpatient and outpatient care, and mortality data.

### Harmonization of medical claims data for the two populations

Our sample selection procedure consisted of three steps to determine population-based age-specific and age-standardized prevalence and establish a cohort-specific state space for sequence and cluster analysis.

**Fig. 1:**
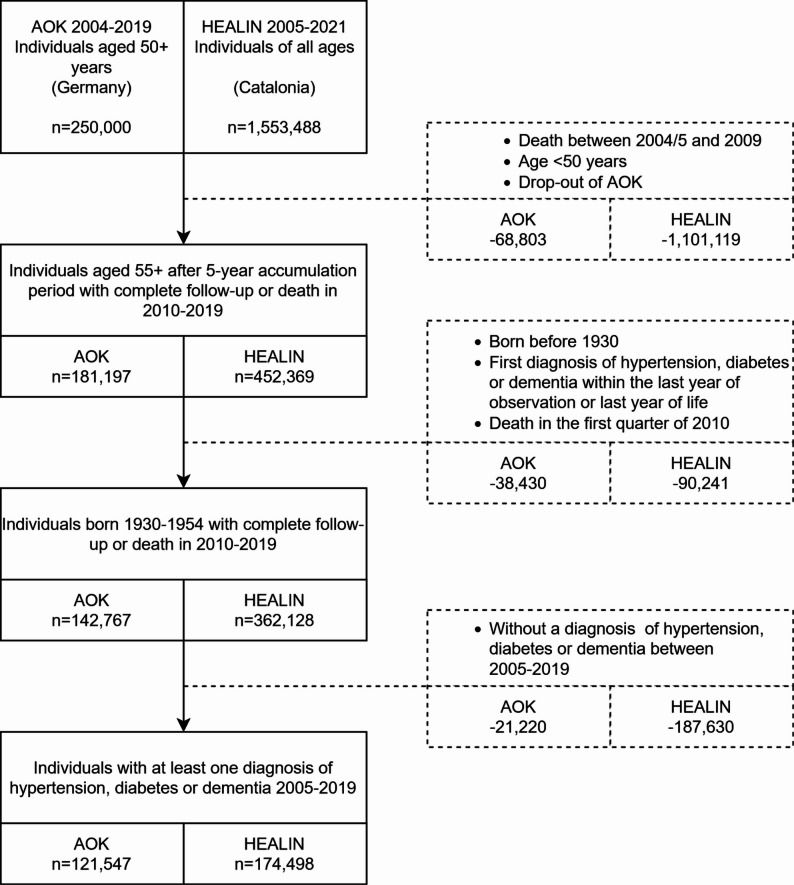
Flow Chart for the Catalan and German analysis samples. Source: HEALIN (2005–2021), AOK (2004–2019)

First, in both populations, the data was harmonized into quarterly health information on individuals from 2005 to the end of 2019. The three morbidities were captured by ICD-10 and included hypertension (I10-I15), diabetes mellitus (E10-E14) and dementia (F00-F03, F05.1, G30, G31.0, G31.82, G23.1). Hypertension, diabetes and dementia were coded as binary “ever” variables, where each condition was assigned a value of one following the first diagnosis and remained zero otherwise. To counteract the left censoring of health information, a five-year accumulation period was introduced during which diagnoses were collected, resulting in an analysis period from 2010 to 2019. In the case of Germany, individuals who left the AOK health insurance were excluded, ensuring that only trajectories with a complete follow-up period or death within the observation period were considered. A diagnosis of hypertension or diabetes was considered valid if it met the M2Q criteria, which requires a minimum of two diagnoses within two quarters across any rolling four-quarter period [33–35]. The identification of dementia cases followed a validation procedure [36]. For Catalonia, diagnoses of hypertension, diabetes and dementia were determined based on recorded disease counts, and a diagnosis was considered valid if it was present in the dataset. These samples of 452,369 Catalans and 181,197 Germans were used for calculating the age-specific and age-standardized prevalence for ages 55 and above in the year 2010 (see Results, Table 1).

Second, we restricted the sequence and cluster analysis to individuals born between 1930 and 1954 to reduce potential bias from left censoring, which is more pronounced among the oldest birth cohorts and may result in false-negative classification of morbid individuals. Accordingly, the analysis samples included individuals 55 to 80 at the beginning of 2010. Additionally, individuals were also excluded if they only received a diagnosis of hypertension, diabetes or dementia within the within the last year of life. Such cases likely reflect irregular healthcare utilization, where conditions remain undocumented until health deteriorates and first contact occurs near death, obscuring the actual preceding morbidity status represented by false-negative diagnosed individuals. In addition, trajectories with a first diagnosis of the three morbidities in the last year of observation were excluded as they were considered as severely right-censored and uninformative for sequence and cluster analysis, resulting in less homogeneous profiles when included. Additionally, individuals were considered non-eligible if they died in the first quarter of observation in 2010. This resulted in 362,128 Catalans and 142,767 Germans.

Third, individuals were included in the sequence and cluster analysis if they had at least one diagnosis of hypertension, diabetes, or dementia between 2005 and the end of 2019 and survived until the second quarter of 2010. The time unit for each state in a trajectory was in quarters. The following nine states were covered in individual trajectories: “No disease” (hereafter, referring to none of the three diseases), “Hypertension”, “Diabetes”, “Hypertension + Diabetes”, “Dementia”, “Dementia + Diabetes”, “Dementia + Hypertension”, “Dementia + Diabetes + Hypertension”, “Death”. In case of death, the individual remained in the state of death until the end of observation. This resulted in 174,498 Catalans and 121,547 Germans (Figure 1).

### Methods

Age-specific prevalence was estimated by summing up the total number of diagnoses in 2010 and dividing them by the population in the first quarter of 2010. For age-standardization, the European Standard Population 2013 [37] was used.

Typologies of morbidity and mortality trajectories in Catalonia and Germany were identified using the CLARA algorithm, a sequence and cluster analysis approach for large datasets [38]. For each population, a distance matrix was computed for a random subsample of 1,000 sequences. The measured distances between all possible pairs of individuals trajectories, also referred to as sequences, was based on the sequence vector representation (SVRspell). The choice of the distance measure is discussed in the following section. Based on the distance matrix, the sequences were clustered using Partitioning Around Medoids (PAM) [39] for two to 12 groups. This clustering algorithms aims to find the best representative sequences (so-called medoids) per each pre-defined number of groups by identifying representative sequences that have the smallest weighted sum of distances to all other sequences in the group. The medoids of each possible group solution were identified and the distances between the medoids and each sequence of the whole sample were computed. Each sequence was assigned to the nearest medoid and based on this, cluster quality indices as well as cluster stability measures were computed. The whole procedure was repeated 500 times to avoid sampling bias. Sensitivity analysis for both the Catalan and the German dataset with larger random sample size (*n* = 2,000) and higher number of iterations (*r* = 1,000) were computed and resulted in similar favorable cluster solutions.

The choice of the distance measure was based on the following rationale: First, the use of timing-sensitive distance measures is not applicable when the process time is in calendar time and not age. This results in individuals having different characteristics in their transition from one state to another. I.e., individuals of younger age have different morbidity characteristics compared to older individuals, particularly in the probability of accumulating more morbidities or dying. Second, a duration-sensitive distance measure is not applicable either, given that the morbidity trajectory of individuals was observed over a relatively short period of 10 years. Individual morbidity in old age deteriorates over a longer period and therefore no conclusions on the actual duration of diseases can be drawn. Ultimately, the decision was made to use an order-sensitive distance measure to explore patterns in the order of hypertension, diabetes and subsequent dementia and death. Subsequence vector representation (SVRspell; with a = 1 and b = 0) was chosen as the distance measure, which have been proved to provide reliable distances sensitive to the order of states elsewhere [38, 40].

The evaluation of cluster quality and cluster stability measures demonstrated that parsimonious cluster solutions were optimal for 10 or more clusters (see Additional file 1, Figures A1a-A2b). An 11-cluster solution for both Catalonia and Germany showed intriguing profiles in terms of similarities and dissimilarities in hypertension, diabetes, dementia and mortality trajectories. More profiles did not yield further insights into morbidity and mortality trajectory patterns, particularly in the context of dementia, which is of central interest in the presented work. For the analysis, the packages “TraMineR” and “WeightedClusters” for RStudio were used [40, 41].

Multinomial logistic regression models were estimated to investigate profile membership characteristics by sex (male, female) and birth cohort (1950–1954, 1945–1949, 1940–1944, 1935–1939, 1930–1934), separately for the Catalan and the German sample. Marginal means were calculated to obtain the adjusted predicted probability for each combination of sex and birth cohort by using the “marginaleffects” package for RStudio [42]. Relative risk estimates can be found in the additional file (Additional file 1, Table A2a & A2b).

## Results

### Basic description of study populations

In 2010, the age-standardized prevalence of hypertension among individuals aged 55 and older was 22.5% (95% confidence interval: 22.2–22.5%) for Catalans and 69.4% (69.0-69.8%) for Germans (Table 1). The prevalence of diabetes was 8.1% (8.0-8.2%) among Catalans and 29.4% (29.2–29.7%) among Germans. For dementia, the prevalence was 2.9% (2.8–2.9%) for Catalans and 8.2% (8.1–8.4%) for Germans. The differences in the prevalence may largely reflect variations in diagnostic recording and data-generation practices rather than genuine morbidity differences. After implementing further harmonization measures to account for data-artifact differences, a total of 174,798 Catalans and 121,547 Germans, born between 1930 and 1954, had at least one diagnosis of hypertension, diabetes, or dementia remained in the analytical sample for the follow-up period between 2010 and 2019 (Table 2). This accounted for 48.3% of all Catalans born between 1930 and 1954 and 85.1% of Germans. The Catalan cohort was generally younger than the German cohort. For instance, the largest proportion of Catalans were born between 1945 and 1949 (22.3%), whereas the majority of Germans were born between 1935 and 1939 (26.1%). The sex distribution was similar in both regions, with 52.3% of the Catalan cohort and 55.0% of the German cohort being women.

**Table 1:** Crude age-specific and age-standardized prevalence age 55 and above in 2010

	Catalonia			Germany		
	No diagnosis	Diagnosis	(Crude) Prevalence in %	No diagnosis	Diagnosis	(Crude) Prevalence in %
**Hypertension**						
**55–59**	73,910	14,757	16.6 (16.4–16.9)	11,141	11,639	51.1 (50.2–52.0)
**60–64**	67,283	17,427	20.6 (20.3–20.9)	9700	15,373	61.3 (60.3–62.3)
**65–69**	53,593	17,064	24.2 (23.8–24.5)	8047	17,946	69.0 (68.0-70.1)
**70–74**	43,957	14,638	25.0 (24.6–25.4)	8472	27,526	76.5 (75.6–77.4)
**75–79**	45,204	15,559	25.6 (25.2–26.0)	5069	23,972	82.5 (81.5–83.6)
**80–84**	34,350	11,746	25.5 (25.0-25.9)	3207	19,136	85.6 (84.4–86.9)
**85–89**	21,223	7162	25.2 (24.6–25.8)	1848	11,765	86.4 (84.9–88.0)
**90+**	11,157	3339	23.0 (22.3–23.8)	998	5358	84.3 (82-86.6)
**55+**	350,677	101,692	22.5 (22.3–22.6)	48,482	132,715	73.2 (72.8–73.6)
**55+ (age-standardized)**			22.4 (22.2–22.5)			69.4 (69.0-69.8)
**Diabetes**						
**55–59**	83,386	5281	6.0 (5.8–6.1)	18,613	4167	18.3 (17.7–18.8)
**60–64**	78,137	6573	7.8 (7.6–7.9)	18,874	6199	24.7 (24.1–25.3)
**65–69**	64,230	6427	9.1 (8.9–9.3)	18,388	7605	29.3 (28.6–29.9)
**70–74**	53,032	5563	9.5 (9.2–9.7)	23,726	12,272	34.1 (33.5–34.7)
**75–79**	55,031	5732	9.4 (9.2–9.7)	18,030	11,011	37.9 (37.2–38.6)
**80–84**	42,180	3916	8.5 (8.2–8.8)	13,642	8701	38.9 (38.1–39.8)
**85–89**	26,254	2131	7.5 (7.2–7.8)	8299	5314	39.0 (38.0-40.1)
**90+**	13,742	754	5.2 (4.8–5.6)	4072	2284	35.9 (34.5–37.4)
**55+**	415,992	36,377	8.0 (8.0-8.1)	123,644	57,553	31.8 (31.5–32.0)
**55+ (age-standardized)**			8.1 (8.0-8.2)			29.4 (29.2–29.7)
**Dementia**						
**55–59**	88,320	347	0.4 (0.4–0.4)	22,484	296	1.3 (1.2–1.4)
**60–64**	84,274	436	0.5 (0.5–0.6)	24,610	463	1.8 (1.7-2.0)
**65–69**	69,947	710	1 0.0 (0.9–1.1)	25,142	851	3.3 (3.1–3.5)
**70–74**	57,268	1327	2.3 (2.1–2.4)	33,972	2026	5.6 (5.4–5.9)
**75–79**	57,784	2979	4.9 (4.7–5.1)	25,706	3335	11.5 (11.1–11.9)
**80–84**	42,209	3887	8.4 (8.2–8.7)	17,751	4592	20.6 (20.0-21.1)
**85–89**	25,107	3278	11.5 (11.2–11.9)	9001	4612	33.9 (32.9–34.9)
**90+**	12,760	1736	12.0 (11.4–12.5)	3182	3174	49.9 (48.2–51.7)
**55+**	437,669	14,700	3.2 (3.2–3.3)	161,848	19,349	10.7 (10.5–10.8)
**55+ (age-standardized)**			2.9 (2.8–2.9)			8.2 (8.1–8.4)

Legend: European Standard Population 2013 was used for age-standardization. Source: HEALIN (2005–2021), AOK (2004–2019)

**Table 2:** Sample descriptions

	Individuals born 1930–1954 *n* (%)			Analysis sample *n* (%)			Share of individuals in analysis sample among individuals born 1930–1954 (%)			
	Women	Men	Total	Women	Men	Total	Women	Men	Total	
**Catalonia**										
**1950-54**	45,249 (12.5)	43,305 (12.0)	88,554 (24.5)	16,701 (9.6)	18,655 (10.7)	35,356 (20.2)	36.9	43.1	39.9	
**1945-49**	43,660 (12.1)	40,888 (11.3)	84,548 (23.3)	19,126 (10.9)	19,915 (11.4)	39,041 (22.3)	43.8	48.7	46.2	
**1940-44**	36,952 (10.2)	33,486 (9.2)	70,438 (19.5)	18,611 (10.6)	17,224 (9.9)	35,835 (20.5)	50.4	51.4	50.9	
**1935-39**	31,623 (8.7)	26,706 (7.4)	58,329 (16.1)	17,249 (9.9)	13,986 (8.0)	31,235 (17.9)	54.5	52.4	53.5	
**1930-34**	34,589 (9.6)	25,670 (7.1)	60,259 (16.6)	19,751 (11.3)	13,580 (7.8)	33,331 (19.1)	57.1	52.9	55.3	
**Total**	192,073 (53.0)	170,055 (47.0)	362,128 (100.0)	91,438 (52.3)	83,360 (47.7)	174,798 (100.0)	47.6	49.0	48.3	
**Germany**										
**1950-54**	14,186 (9.9)	13,724 (9.6)	27,910 (19.5)	10,419 (8.6)	10,414 (8.6)	20,833 (17.1)	73.4	75.9	74.6	
**1945-49**	11,935 (8.4)	11,432 (8.0)	23,367 (16.4)	9675 (8.0)	9375 (7.7)	19,050 (15.7)	81.1	82.0	81.5	
**1940-44**	15,145 (10.6)	13,850 (9.7)	28,995 (20.3)	13,190 (10.9)	11,900 (9.8)	25,090 (20.6)	87.1	85.9	86.5	
**1935-39**	19,959 (14.0)	15,394 (10.8)	35,353 (24.8)	18,176 (15.0)	13,556 (11.2)	31,732 (26.1)	91.1	88.1	89.8	
**1930-34**	16,593 (11.6)	10,549 (7.4)	27,142 (19.0)	15,429 (12.7)	9413 (7.7)	24,842 (20.4)	93.0	89.2	91.5	
**Total**	77,818 (54.5)	64,949 (45.5)	142,767 (100.0)	66,889 (55.0)	54,658 (45.0)	121,547 (100.0)	86.0	84.2	85.1	

Legend: Sample sizes corresponding to individuals eligible for follow-up 2010–2019. Sources: HEALIN (2005–2021), AOK (2004–2019)

### Description of morbidity and mortality trajectory profiles

**Fig. 2:**
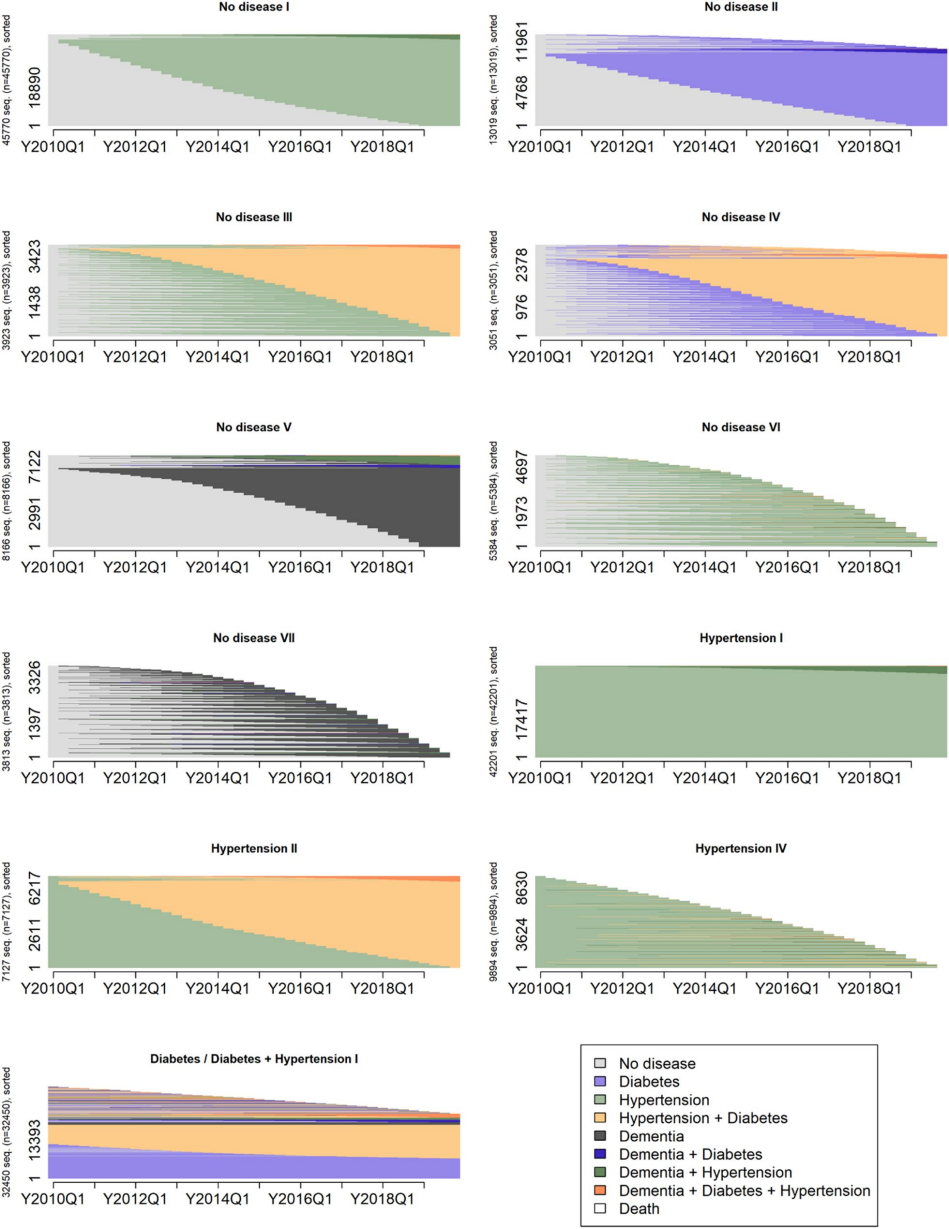
Catalan morbidity and mortality trajectory profiles. Index plot. No disease = None of the three diseases. Source: HEALIN (2005–2021)

**Fig. 3:**
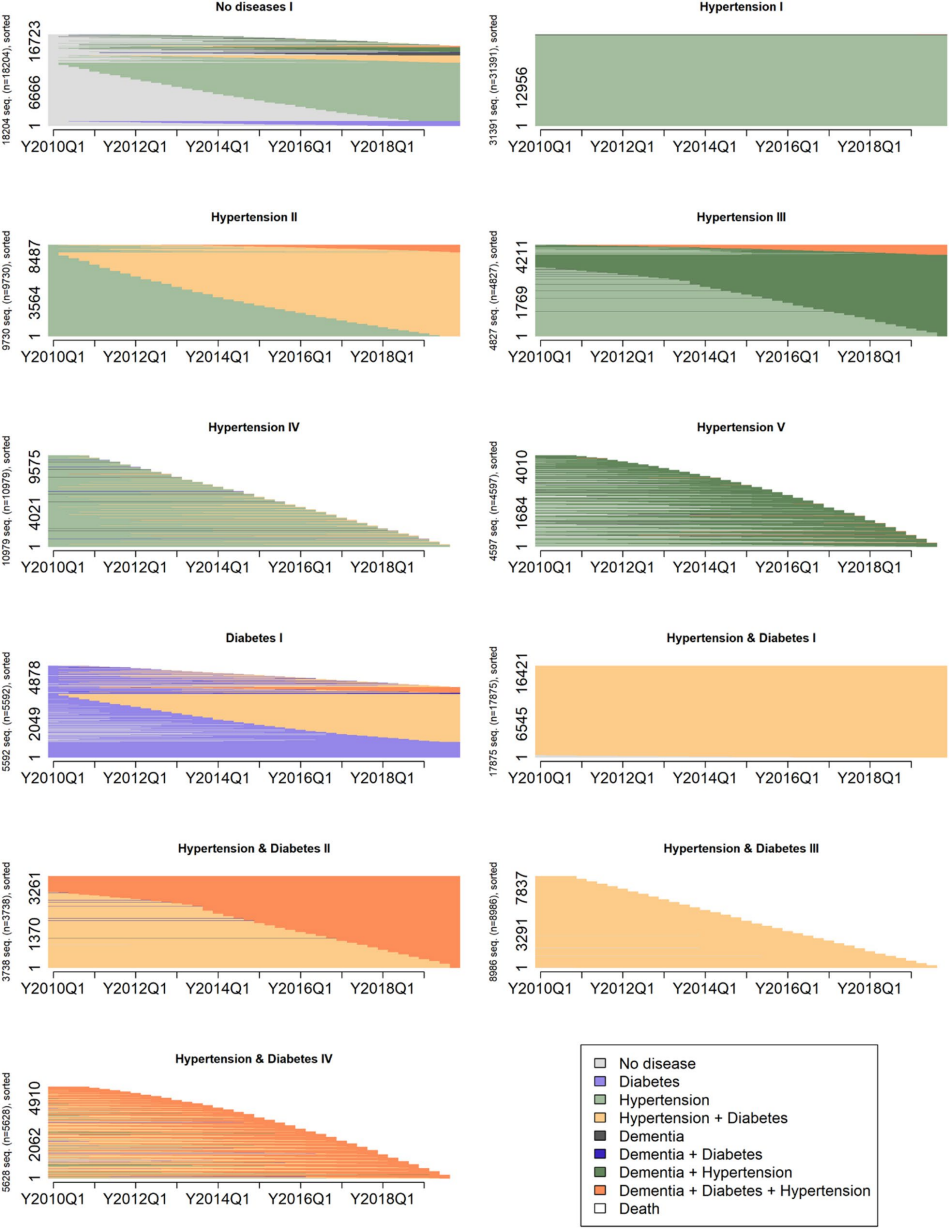
German morbidity and mortality trajectory profiles. Index plot. No disease = None of the three diseases. Source: AOK (2004–2019)

We found diverse hypertension, diabetes, dementia and mortality trajectories. In order to reduce the complexity of the dissimilarities and similarities between individual trajectories, we compared them based on the ordering of states by using the subsequence vector representation (SVRspell) and sorted them into profiles based on the most representative trajectories (Partitioning Around Medoids). This approach yielded 11 distinct morbidity and mortality profiles, both for the Catalan and German population (see Additional file 1, Figure A1a-A2b). When the profiles were organized according to their starting states, the dissimilarities and similarities in the characteristics became more evident (Figs. 2 and 3).

#### Starting from no disease

In Catalonia, seven profiles initially had none of the three diseases (No disease I-VII), compared to only one such profile in Germany (No disease I) (Table 3). The most common trajectory in this profile involved a progression from no disease to hypertension, a profile present in both populations but considerably more prevalent in Catalonia (26.2%) than in Germany (15.0%). Among the six additional Catalan profiles, one was defined by a progression to diabetes (No disease II, 7.4%) and another by a progression to dementia (No disease V, 4.7%). A third profile experienced a rapid transition to hypertension, followed by the combination of hypertension and diabetes (No disease III, 2.2%), while a fourth initially developed diabetes, later followed by a combination of diabetes and hypertension (No disease IV, 1.7%). The remaining two profiles were marked by the onset of either hypertension (No disease VI, 3.1%) or dementia (No disease VII, 2.2%), both followed by a rapid progression to death.

**Table 3:** Profile characteristics

From state…		Catalonia			Germany		
		To state(s)…	Dynamic	%	To state(s)…	Dynamic	%
**No disease**	**I**	HTN	slow	26.2	HTN	slow	15.0
	**II**	DM	slow	7.4			
	**III**	HTN → HTN + DM	rapid	2.2			
	**IV**	DM → DM + HTN	rapid	1.7			
	**V**	DEM	slow	4.7			
	**VI**	HTN → Death	rapid	3.1			
	**VII**	DEM → Death	rapid	2.2			
**Hypertension**	**I**	HTN	persistent	24.1	HTN	persistent	25.8
	**II**	HTN + DM	slow	4.1	HTN + DM	slow	8.0
	**III**				HTN + DEM	slow	4.0
	**IV**	Death	slow	5.7	Death	slow	9.0
	**V**				HTN + DEM → Death	rapid	3.8
**Diabetes**	**I**				DM + HTN / Death	mixed	4.6
**Diabetes / Diabetes & Hypertension**	**I**	DM / DM + HTN / Death	mixed	18.6			
**Hypertension & Diabetes**	**I**				HTN + DM	persistent	14.7
	**II**				HTN + DM+DEM	slow	3.1
	**III**				Death	slow	7.4
	**IV**				HTN + DM+DEM → Death	rapid	4.6

Legend: HTN = Hypertension; DM = Diabetes mellitus; DEM = Dementia; slow = majority of trajectories with one state transition; rapid = majority of trajectories with at least two state transitions; persistent: majority of trajectory without state transition; mixed = diverse trajectories. Sources: HEALIN (2005–2021), AOK (2004–2019)

#### Starting from hypertension

Three Catalan profiles initially experienced hypertension, whereas in Germany, five such profiles existed. All three profiles present in Catalonia were also observed in Germany. Both populations included a profile with persistent hypertension only (Hypertension I: Catalonia 24.1%, Germany 25.8%). A second shared profile exhibited a progression to a combination of hypertension and diabetes (Hypertension II), though its prevalence was twice as high in Germany (8.0%) as in Catalonia (4.1%). A third common profile was characterized by a progression to death (Hypertension IV: Catalonia 5.7%, Germany 9.0%). Two additional profiles were unique to Germany: one progressed to the combination of hypertension and dementia (Hypertension III, 4.0%), while the other exhibited a rapid transition to dementia followed by death (Hypertension V, 3.8%).

#### Starting from diabetes

In Germany, one additional heterogeneous profile was identified, broadly characterized by initial diabetes with subsequent persistence of the disease or progression to the combination of diabetes and hypertension at varying rates, also with some trajectories culminating in death (Diabetes I, 4.6%).

#### Starting from diabetes, or diabetes and hypertension

A heterogeneous profile was observed in Catalonia, with the majority of individuals being characterized by persistent diabetes or a combination of diabetes and hypertension (Diabetes / Diabetes & Hypertension I, 18.6%). Approximately one quarter of individuals within this profile followed more diverse trajectories, most culminating in death, frequently preceded by dementia or dementia-related morbidity combinations. A subset of these trajectories was characterized by initial dementia at the start of observation.

#### Hypertension and diabetes

Four German profiles existed with trajectories starting with a combination of hypertension and diabetes; however, no equivalent profile was observed in Catalonia. The largest of these profiles consisted of individuals with persistent hypertension and diabetes combined (Hypertension & Diabetes I, 14.7%). The remaining three profiles exhibited a progression to dementia (Hypertension & Diabetes II, 3.1%), a progression to death (Hypertension & Diabetes III, 7.4%), or a rapid progression to dementia followed by death (Hypertension & Diabetes IV, 4.6%).

### Morbidity and mortality trajectory profile differences by birth cohort and sex

#### Differences by birth cohorts

**Fig. 4:**
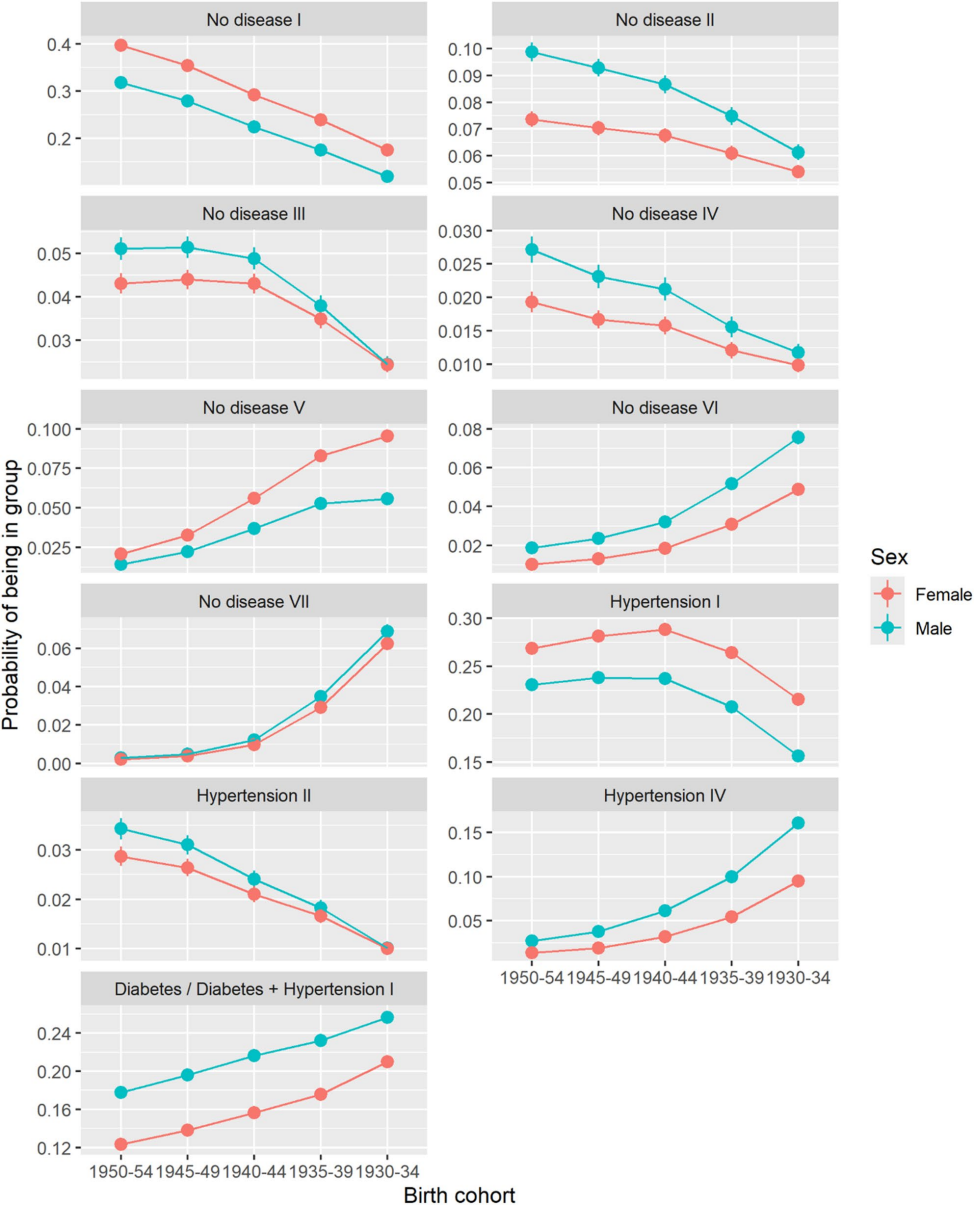
Adjusted probability of being in Catalan profile. Variable y-scale. Source: HEALIN (2005–2021)

**Fig. 5:**
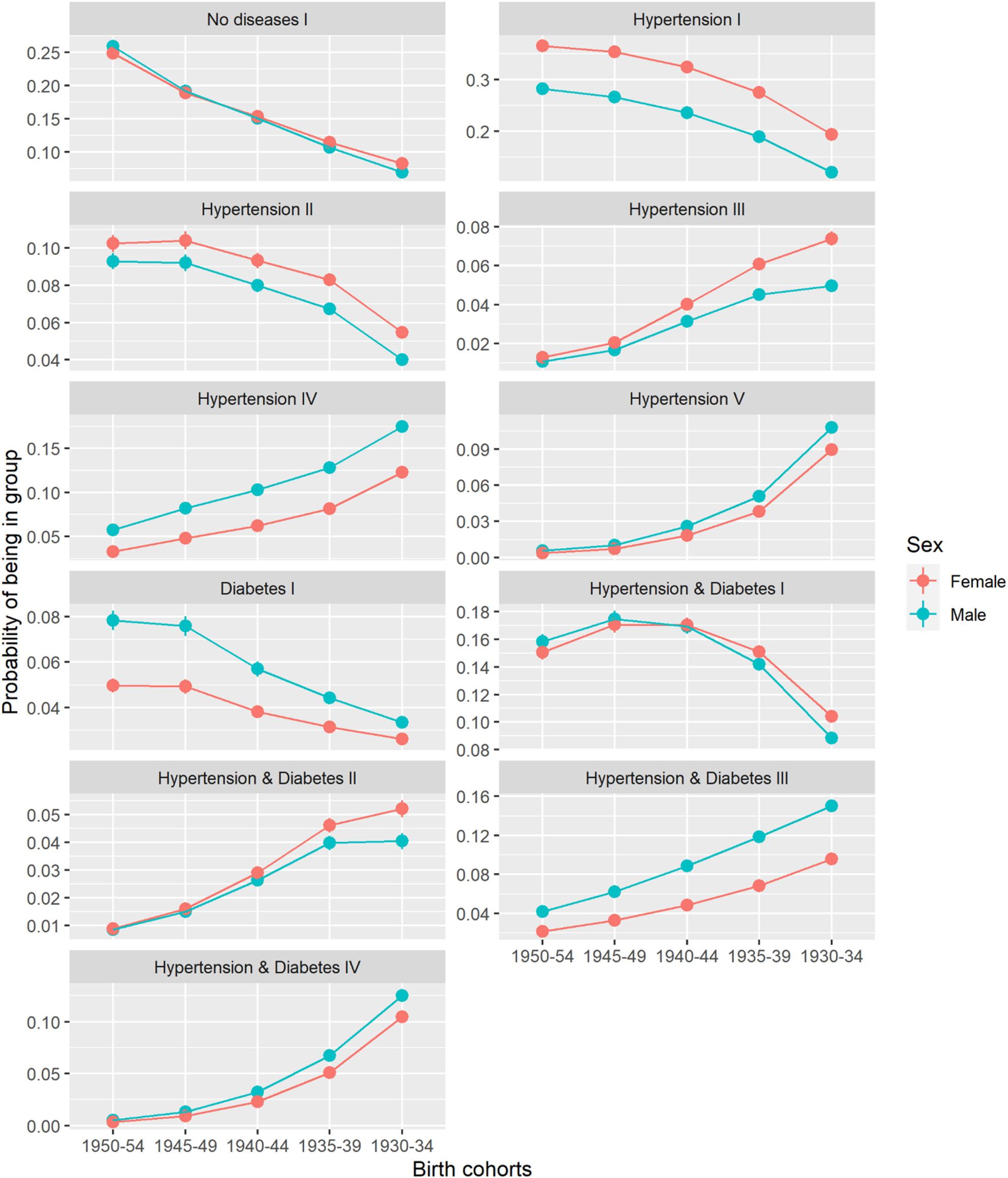
Adjusted probability of being in German profile. Variable y-scale. Source: AOK (2004–2019)

Morbidity and mortality trajectory profiles varied across birth cohorts in both Catalonia and Germany (Figs. 4 and 5; Tables 4 and 5; Additional file 1, Table A2a & A2b). In Catalonia, younger birth cohorts were more likely to progress from no disease to hypertension or diabetes. For instance, Catalan men born between 1950 and 1954 were the most likely to progress from no disease to diabetes (No disease II, *p* = 0.099, 95% confidence interval: [0.095–0.102]), to hypertension followed by diabetes (No disease III, *p* = 0.051 [0.049–0.054]), and to diabetes followed by hypertension (No disease IV, *p* = 0.027 [0.025–0.029]). This trend weakened in older cohorts. In Germany, younger birth cohorts demonstrated a higher probability of persistently living with hypertension (Hypertension I), a combination of hypertension and diabetes (Hypertension & Diabetes I), or progressed from no disease (No disease I) or at least one disease (Hypertension II, Diabetes I) to more severe morbidity stages. For instance, German women (*p* = 0.249 [0.242–0.256]) and men (*p* = 0.259 [0.253–0.266]) born between 1950 and 1954 had the highest probability to progress from no disease to hypertension (No disease I) compared to older birth cohorts. Both in Catalonia and Germany, older birth cohorts were more likely to progress to dementia, to a higher number of morbidity combinations, or to death. For instance, both in Catalonia (*p* = 0.161 [0.156–0.166]) and Germany (*p* = 0.175 [0.169–0.181]), men born between 1930 and 1934 were most likely to progress from hypertension to death (Hypertension IV). Both Catalan and German women were more likely to be in a persistent state of hypertension (Hypertension I). However, in this profile Catalan women were most likely born between 1940 and 1944 (*p* = 0.289 [0.283–0.294]), while German women were most likely born between 1950 and 1954 (*p* = 0.365 [0.358–0.372]).

**Table 4:** Predicted probability to be in trajectory profile by birth cohort and sex, Catalan population

Marginal effect (95% CI)	No disease I*	No disease II	No disease III	No disease IV	No disease V	No disease VI	No disease VII	Hypertension I*	Hypertension II*	Hypertension IV*	Diabetes / Diabetes + Hypertension I
**Male**											
1950-54	0.318	0.099	0.051	0.027	0.014	0.019	0.003	0.231	0.034	0.027	0.178
	(0.312–0.323)	(0.095–0.102)	(0.049–0.054)	(0.025–0.029)	(0.013–0.015)	(0.017–0.020)	(0.002–0.004)	(0.226–0.235)	(0.032–0.036)	(0.025–0.029)	(0.173–0.182)
1945-49	0.279	0.093	0.051	0.023	0.022	0.024	0.005	0.238	0.031	0.038	0.196
	(0.274–0.284)	(0.090–0.096)	(0.049–0.054)	(0.021–0.025)	(0.021–0.024)	(0.022–0.025)	(0.004–0.006)	(0.233–0.243)	(0.029–0.033)	(0.036–0.040)	(0.191-0.200)
1940-44	0.223	0.087	0.049	0.021	0.037	0.032	0.012	0.237	0.024	0.061	0.216
	(0.219–0.228)	(0.083–0.090)	(0.046–0.051)	(0.020–0.023)	(0.035–0.039)	(0.030–0.034)	(0.011–0.014)	(0.232–0.242)	(0.022–0.026)	(0.058–0.064)	(0.211–0.221)
1935-39	0.175	0.075	0.038	0.016	0.053	0.052	0.035	0.208	0.018	0.100	0.232
	(0.171–0.179)	(0.072–0.078)	(0.036–0.040)	(0.014–0.017)	(0.050–0.055)	(0.049–0.055)	(0.032–0.037)	(0.203–0.212)	(0.017–0.020)	(0.095–0.104)	(0.227–0.237)
1930-34	0.118	0.061	0.024	0.012	0.056	0.076	0.069	0.156	0.010	0.161	0.256
	(0.115–0.122)	(0.058–0.064)	(0.023–0.026)	(0.010–0.013)	(0.053–0.059)	(0.072–0.079)	(0.065–0.072)	(0.152–0.161)	(0.009–0.011)	(0.156–0.166)	(0.251–0.262)
**Female**											
1950-54	0.397	0.074	0.043	0.019	0.021	0.010	0.002	0.269	0.029	0.013	0.123
	(0.391–0.403)	(0.071–0.077)	(0.041–0.045)	(0.018–0.021)	(0.019–0.022)	(0.009–0.011)	(0.002–0.003)	(0.264–0.274)	(0.027–0.031)	(0.012–0.014)	(0.120–0.127)
1945-49	0.354	0.070	0.044	0.017	0.033	0.013	0.004	0.282	0.026	0.019	0.138
	(0.349–0.360)	(0.068–0.073)	(0.042–0.046)	(0.015–0.018)	(0.031–0.035)	(0.012–0.014)	(0.003–0.004)	(0.276–0.287)	(0.025–0.028)	(0.018–0.021)	(0.134–0.142)
1940-44	0.291	0.068	0.043	0.016	0.056	0.018	0.010	0.289	0.021	0.032	0.156
	(0.286–0.297)	(0.065–0.070)	(0.041–0.045)	(0.014–0.017)	(0.053–0.059)	(0.017–0.020)	(0.009–0.011)	(0.283–0.294)	(0.019–0.023)	(0.030–0.034)	(0.153–0.160)
1935-39	0.238	0.061	0.035	0.012	0.083	0.031	0.029	0.264	0.017	0.054	0.176
	(0.233–0.244)	(0.058–0.064)	(0.033–0.037)	(0.011–0.013)	(0.079–0.087)	(0.029–0.033)	(0.027–0.031)	(0.259–0.269)	(0.015–0.018)	(0.052–0.057)	(0.171–0.180)
1930-34	0.174	0.054	0.024	0.010	0.095	0.049	0.062	0.215	0.010	0.095	0.210
	(0.170–0.179)	(0.052–0.057)	(0.023–0.026)	(0.009–0.011)	(0.092–0.099)	(0.046–0.051)	(0.059–0.065)	(0.210–0.220)	(0.009–0.011)	(0.092–0.099)	(0.205–0.215)

Legend: * also in German population; 95% confidence interval (CI) in parenthesis. Source: HEALIN (2005–2021)

**Table 5:** Predicted probability to be in trajectory profile by birth cohort and sex, German population

Marginal effect (95% CI)	No disease I*	Hypertension I*	Hypertension II*	Hypertension III	Hypertension IV*	Hypertension V	Diabetes I	Hypertension & Diabetes I	Hypertension & Diabetes II	Hypertension & Diabetes III	Hypertension & Diabetes IV
**Male**											
1950-54	0.259	0.282	0.093	0.011	0.057	0.006	0.078	0.158	0.009	0.042	0.005
	(0.253–0.266)	(0.275–0.288)	(0.089–0.097)	(0.009–0.012)	(0.054–0.061)	(0.005–0.007)	(0.074–0.083)	(0.153–0.164)	(0.007–0.010)	(0.039–0.045)	(0.004–0.006)
1945-49	0.192	0.266	0.092	0.017	0.082	0.010	0.076	0.175	0.015	0.062	0.013
	(0.186–0.198)	(0.259–0.272)	(0.088–0.096)	(0.015–0.018)	(0.078–0.087)	(0.009–0.012)	(0.071–0.080)	(0.169–0.181)	(0.013–0.017)	(0.058–0.066)	(0.011–0.015)
1940-44	0.151	0.235	0.080	0.031	0.103	0.026	0.057	0.169	0.026	0.089	0.032
	(0.146–0.156)	(0.230–0.241)	(0.076–0.084)	(0.029–0.034)	(0.099–0.108)	(0.024–0.028)	(0.054–0.060)	(0.164–0.174)	(0.024–0.029)	(0.084–0.093)	(0.030–0.035)
1935-39	0.107	0.189	0.067	0.045	0.128	0.051	0.044	0.142	0.040	0.119	0.067
	(0.103–0.111)	(0.184–0.194)	(0.064–0.070)	(0.043–0.048)	(0.124–0.133)	(0.048–0.054)	(0.042–0.047)	(0.137–0.146)	(0.037–0.042)	(0.114–0.123)	(0.064–0.071)
1930-34	0.069	0.120	0.040	0.050	0.175	0.108	0.033	0.088	0.040	0.150	0.125
	(0.066–0.073)	(0.116–0.125)	(0.038–0.043)	(0.046–0.053)	(0.169–0.181)	(0.103–0.113)	(0.031–0.036)	(0.084–0.092)	(0.038–0.043)	(0.144–0.156)	(0.120–0.131)
**Female**											
1950-54	0.249	0.365	0.102	0.013	0.032	0.004	0.050	0.151	0.009	0.022	0.004
	(0.242–0.256)	(0.358–0.372)	(0.098–0.107)	(0.011–0.015)	(0.030–0.035)	(0.003–0.005)	(0.047–0.053)	(0.145–0.156)	(0.008–0.010)	(0.020–0.023)	(0.003–0.004)
1945-49	0.189	0.354	0.104	0.020	0.048	0.007	0.049	0.171	0.016	0.033	0.009
	(0.183–0.195)	(0.346–0.361)	(0.099–0.109)	(0.018–0.023)	(0.045–0.051)	(0.006–0.008)	(0.046–0.052)	(0.165–0.176)	(0.014–0.018)	(0.031–0.035)	(0.008–0.010)
1940-44	0.154	0.324	0.093	0.040	0.062	0.018	0.038	0.171	0.029	0.048	0.023
	(0.149–0.158)	(0.317–0.330)	(0.089–0.097)	(0.037–0.043)	(0.059–0.065)	(0.017–0.020)	(0.036–0.041)	(0.165–0.176)	(0.027–0.031)	(0.046–0.051)	(0.021–0.025)
1935-39	0.115	0.274	0.083	0.061	0.081	0.038	0.031	0.151	0.046	0.068	0.051
	(0.111–0.119)	(0.269–0.280)	(0.080–0.086)	(0.058–0.064)	(0.078–0.085)	(0.036–0.040)	(0.029–0.033)	(0.147–0.155)	(0.044–0.049)	(0.065–0.071)	(0.048–0.054)
1930-34	0.082	0.194	0.055	0.074	0.123	0.090	0.026	0.104	0.052	0.096	0.105
	(0.079–0.086)	(0.188–0.199)	(0.052–0.058)	(0.070–0.078)	(0.119–0.127)	(0.086–0.094)	(0.024–0.028)	(0.100-0.108)	(0.049–0.055)	(0.092–0.099)	(0.101–0.109)

Legend: * also in Catalan population; 95% confidence interval (CI) in parenthesis. Source: AOK (2004–2019)

#### Differences by sex

General sex differences between morbidity and mortality trajectory profiles existed in Catalonia and Germany. Across both populations, especially women of older birth cohorts were most likely to progress from varying states to dementia, while older men were more likely to rapidly progress to death. E.g., probability of progressing from no disease to dementia was higher for Catalan women born between 1930 and 1934 (No disease V, *p* = 0.095 [0.092–0.099]), compared to their male counterparts (No disease V, *p* = 0.056 [0.053–0.059]). Women were more likely to be characterized by a longer duration of hypertension only, while profiles mainly characterized by diabetes were dominated by men. For instance, German men born between 1930 and 1934 (Hypertension & Diabetes III, *p* = 0.150 [0.144–0.156]) were most likely to progress from a combination of hypertension and diabetes to death, compared to German females of the same birth cohort (Hypertension & Diabetes III, *p* = 0.096 [0.092–0.099]).

## Discussion

In this study, we aimed to compare morbidity and mortality trajectories of hypertension, diabetes, and dementia using administrative medical data from the two structurally distinct healthcare systems of Catalonia and Germany. Through this cross-population comparative approach, we sought to elucidate the insights that can be derived from analyzing administrative medical data. The objectives of this study were threefold: first, to determine the number of individuals with hypertension, diabetes and dementia; second, to characterize morbidity and mortality trajectory profiles by sequence and cluster analysis for large datasets, and to examine whether similar profiles emerged in Catalonia and Germany; and third, to investigate how these trajectory profiles varied by birth cohort and sex.

### Principal findings

We identified profound differences in the prevalence of hypertension, diabetes, and dementia between Catalonia and Germany. At the start of the observation period in 2010, all three conditions and their combinations were less common in Catalonia than in Germany. The trajectory profiles showed considerable variability, with most Catalans initially free from any of the three morbidities, whereas Germans were more likely to begin with hypertension. Overall, Catalan individuals experienced fewer morbidity combinations and were typically diagnosed with a single condition of interest, while German individuals had prolonged exposure to these morbidities, including their combinations. No common profile defined by dementia as the primary morbidity was shared across both populations. In both populations, women were more frequently observed in profiles characterized by a progression to dementia, whereas men were more often facing a deterioration to death, including trajectories preceded by dementia. Younger birth cohorts dominated profiles starting with no disease or hypertension, whereas older birth cohorts were more present in profiles ending with dementia or death. Although we found Catalan and German individuals with similar morbidity and mortality trajectory profiles, these individuals partly differed in their demographic characteristics and the profiles differed by their size.

### Rationale for cross-population comparison

Several similarities between the two data sources formed the rationale for conducting a comparative study. Catalonia and Germany maintain mandatory healthcare coverage systems, ensuring large proportions of the general population are covered in administrative medical data. Both datasets provided a long follow-up period from 2005 to 2019, with the possibility to analyze morbidity trajectories at quarterly intervals. Diagnostic information from both ambulatory and stationary care settings, including institutionalized individuals, was available in both datasets. The diagnosis history was based on the ICD-10 coding system, and both datasets contained information on demographics and mortality. When limiting both samples to individuals aged 50 years and above over an observation period from 2005 to 2019, both datasets were affected by left truncation as well as left and right censoring.

### Data harmonization

We identified five domains for which harmonization was needed, and we were able to address three of them. Firstly, in both data sources, the left censoring of morbidity information was accounted for by establishing an accumulation period of five years, in which individuals were able to receive diagnoses. Moreover, we excluded individuals born before 1930, as we expected that the problem of left censoring was more severe among the oldest old. Secondly, we aimed to identify false-negatively diagnosed individuals who followed irregular healthcare utilization patterns by excluding those who received the first diagnoses of the three morbidities in their final year of life in both the Catalan and German data. In addition, we limited the analysis to individuals with at least one of the three morbidities diagnosed and excluded all others. Thirdly, we excluded trajectories that were severely right-censored, i.e., those that only received a first diagnosis of the three morbidities during the last year of observation. The two domains we were not able to account for were, first, the exclusion of false-positive diagnoses. In the German data, we implemented the M2Q criterion for hypertension and diabetes and an internal validation procedure for dementia. In the Catalan data, we relied on the initial recording of diagnosis, which was recorded upon clinical confirmation of a chronic disease, and no further validation was performed. Second, we excluded individuals who dropped out of the AOK sample (8.7%) from our analysis, while we were not able to identify individuals who emigrated from the Catalan cohort, i.e., left the healthcare system. However, migration rates among older residents in Catalonia were low. In 2019, fewer than four per 1,000 inhabitants aged 50–54 moved to another Spanish region, declining to fewer than three per 1,000 among those aged 75 and above. Emigration to foreign destinations was similarly rare, at fewer than nine and two per 1,000 for the same age groups, respectively [43, 44].

### Description of differences between the two populations

After the implementation of the harmonization measures, only 48.3% of Catalans but 85.1% of Germans remained in the final analysis samples. The proportion of younger birth cohorts among Catalans was higher, while fewer older birth cohorts were included than in the German population. The main reason for this might be that hypertension and diabetes most often occur in midlife [45, 46] and therefore the first diagnosis is missing in the Catalan data due to left censoring. These patterns in the age structure of the samples suggest data artifacts that arise during the data generation process, leading to differential extents of left censoring: We assume that older Catalans have received relevant diagnoses prior to 2005 and consequently appeared without diagnoses in the observation period. In Catalonia, diagnoses are typically recorded at the initial consultation, whereas German coding practices require the recording of diagnoses each time clinical symptoms manifest and treatment continues. In addition, in Germany, qualified medical doctors may receive a surcharge for the screening for chronic diseases such as hypertension, which may result in disproportionate diagnosis recording. To counteract the latter, we implemented the widely established M2Q criterion [33–35] and the validation procedure for dementia [36].

However, some differences may reflect genuine variations between the populations. The prevalence of all three conditions in Catalonia appeared to be lower than reported in other studies [8, 47], while the German prevalence was higher than in comparable studies [9, 47]. Direct comparison with reference studies was limited by differences in time periods and age ranges. In the German sample, individuals from Eastern German states were slightly overrepresented. In these states, for instance, hypertension prevalence is higher compared to Western German states [48]. This might explain the higher share of hypertension-dominant trajectories observed in the German population.

Sequence and cluster analysis revealed that Catalan individuals were more often only recently diagnosed with one of the morbidities, whereas in Germany, individuals had often been living with these conditions since the beginning of the follow-up period. The German population also had a higher proportion of older individuals, which may explain the greater share of morbidity profiles that began with an existing morbidity or a combination of morbidities. The longer individuals live, the more morbidities they are likely to accumulate. Furthermore, this finding is reinforced by the association between higher mortality and severe morbidity [49], which aligns with the lower life expectancy observed in Germany compared to Catalonia.

Social inequality within and between Catalonia and Germany may exist and may contribute to differences in the occurrence of hypertension, diabetes, dementia, and mortality [50–52]. In terms of dementia, one possible explanation might be a socioeconomic gradient in dementia risk and healthcare coverage. Evidence suggests that individuals with lower education face a higher dementia risk [12, 53, 54]. Populations covered by statutory insurance or public healthcare may be more likely to have lower education compared to those using private healthcare. While in Catalonia every individual independent of their socioeconomic status is eligible for public healthcare, the AOK specifically is known to insure individuals with lower income and lower occupational complexity [55], resulting in poorer health outcomes in general [56].

The observed sex difference, that women more frequently experienced a progression to dementia and men a deterioration to death, including trajectories preceded by dementia, may occur due to differences in the frequency of practitioner consultations. Dementia may develop unnoticed in the last stage of life, which may result in fewer consultations and recorded diagnoses, particularly among men, while death is always documented. In addition, men more often face fatal diseases, resulting in earlier death and less time with morbidities, while women are prone to less fatal conditions [57], leading to increased time spent in morbidity and with morbidity combinations.

### Implications for comparative studies

Comparing health trajectories across populations using routinely collected administrative medical data is feasible but requires careful consideration of differences between healthcare systems, particularly in the data-generation processes and diagnostic recording practices. Descriptive analytical tools such as prevalence estimates as well as sequence and cluster analysis can reveal cross-system differences in morbidity and mortality trajectories. However, variation in the extent of left censoring is likely the main challenge in distinguishing true morbidity differences from data artifacts. In the present study, some patterns may reflect genuine differences, as indicated by younger birth cohorts predominating profiles starting with no disease or hypertension and older birth cohorts in those ending with dementia or death.

Future comparative research should focus on further harmonizing administrative data to develop healthcare system–adapted minimum standards for studying life-course morbidity. Attention should first be directed towards identifying healthcare system-level differences, particularly the system-specific regularities under which diagnoses are made, shaping diagnostic recording frequency and practices. In Catalonia, diagnoses of chronic conditions are typically recorded at initial consultation, whereas in Germany they are documented more frequently, often as a prerequisite for treatment or medication. Evidence from a Catalan study using medical records suggests additional discrepancies: for example, about 11% of nearly 10,000 individuals treated for dementia received antidementia medication without a recorded diagnosis [17]. While a European guideline for hypertension management is established and regularly updated [58] and a guideline for diabetes management is under development [59], diagnostic standards for dementia may still differ across Europe, despite efforts to establish an early diagnosis standard [60]. Consequently, future research should systematically address both under- and overreporting of diagnoses.

Beyond healthcare system-level factors, differences in how individuals interact with healthcare systems also warrant consideration. Public awareness of diseases, particularly for dementia [61], may influence whether conditions remain unnoticed or receive more attention, leading to incomplete or inflated recording in medical records. Healthcare-seeking behavior, patient-physician interactions, and the accessibility and availability of services [62] may further introduce systematic bias, particularly when these factors vary systematically along socioeconomic or spatial gradients. For instance, Catalonia is characterized by a higher degree of urbanization than Germany, with a high share of individuals concentrated in the urban region of Barcelona. This urban-rural difference may reflect disparities in healthcare accessibility between the two populations.

After accounting for these sources of bias, genuine variations in morbidity trajectories may be attributable to social, economic, and environmental factors, or population dynamics. Ideally, administrative medical data should meet minimal requirements such as similar diagnostic standards, unified coding systems, and comparable follow-up intervals, and should include complete medical histories from birth to minimize left censoring and left truncation. Linking medical prescription data would further strengthen disease validation and assessment of the course of disease.

### Strengths and limitations

To the best of our knowledge, this is the first comparative study on morbidity and mortality trajectories of two European populations, employing longitudinal administrative medical claims data from two differing healthcare systems. By estimating prevalence and using sequence and cluster analysis for large datasets, we investigated individual morbidity and mortality trajectories, which enabled us to determine which insights can be derived from cross-population comparisons based on administrative data. There is no recall bias regarding the history of individual morbidity trajectories, and no selection bias by type of residence was present, as institutionalized individuals were covered in the data. There was no self-selection into the study population, as all individuals were eligible for the study, irrespective of their health status.

The analysis of medical claims data did not allow for the determination of the actual onset of the diseases under study. Therefore, initial symptoms may have occurred prior to the recorded diagnosis. In addition, changes in diagnosis over time, such as for hypertension, and missing or incorrect coding of diagnosis may have occurred, especially for dementia. However, we implemented a validation strategy for Germany to overcome misclassification [36], and in Catalonia, validity has been assessed in an earlier study also based on data from the Catalan Health Institute [8]. Moreover, both data sources are lacking information on individual socioeconomic indicators, which may have been beneficial for distinguishing between genuine differences in morbidity and data artifacts. A notable limitation of the present study is its focus on only two risk factors, hypertension and diabetes, and their trajectories with subsequent dementia and mortality. Other risk factors covered in medical claims data could have been addressed, including depression, hearing loss, high low-density lipoprotein (LDL) cholesterol, traumatic brain injury, obesity or visual loss [12]. However, an increased number of risk factors could have resulted in highly complex trajectories, limiting the generalizability and interpretability of conclusions. In addition, men and women differ in the prevalence of dementia risk factors and their combinations [14, 16, 24, 25]. Results may be biased by focusing on diabetes, which is more prevalent in men with dementia in both Catalonia [24] and Germany [25]. Future research could address this by identifying sex-specific risk factors and analyzing men and women separately. Methodologically, a recognized disadvantage of sequence analysis is the alignment of rare trajectories to clusters that do not reflect the main characteristics of such rare trajectories. This is somewhat arbitrary, as these trajectories often show similarities to more than one cluster. This impedes the interpretability of single profiles. The fuzzy clustering approach might counteract this circumstance, allowing groups to overlap and to align rare trajectories to more than just one cluster [38].

## Conclusion

Our study revealed significant differences between the two European populations — Catalonia and Germany — in the prevalence as well as the morbidity and mortality trajectories of hypertension, diabetes, and dementia. Catalans exhibited more favorable health outcomes, with lower overall morbidity prevalence and less deterioration in morbidity trajectories compared to Germans. These findings demonstrate that cross-population comparison of morbidity trajectories using administrative health data is feasible. Mandatory coverage systems, long and consistent follow-up periods, detailed medical information, and inclusion of vulnerable population groups, which are rarely represented in other data sources for observational studies, provide unique opportunities for population-level longitudinal research.

However, our analysis also demonstrated that such comparisons require careful consideration of healthcare system differences, particularly in diagnostic recording and data-generation practices. While harmonization strategies can address some sources of bias, others remain difficult to eliminate. Differential left censoring emerged as the principal challenge in distinguishing genuine morbidity trajectory differences from data artifacts.

To leverage the potential of a European health data space for comparative morbidity research, further efforts are needed to develop healthcare system-adapted minimum standards, systematically document diagnostic recording practices, and link medical prescription data to strengthen disease validation. Ideally, complete medical histories from birth should be made available to minimize left censoring and left truncation. Once researchers account for these methodological challenges, administrative health data can serve as a powerful resource for understanding morbidity trajectories across countries.

## Supplementary Information

Below is the link to the electronic supplementary material.

Supplementary Material 1.

## Data Availability

The data that support the findings of this study are available from the Catalan Health Institute (HEALIN cohort) and the Scientific Research Institute of the Allgemeine Ortskrankenkasse (AOK), WIdO, but restrictions apply to the availability of these data, which were used under license for the current study, and so are not publicly available. Data, however, can be requested. While the HEALIN cohort data is not currently accessible on a public server, we encourage collaborative ventures that undertake complementary data analyses, cross-national comparisons, and innovative methodological approaches to chronic disease and multimorbidity research. For additional information, please contact Iñaki Permanyer ( [ipermanyer@ced.uab.es](mailto: ipermanyer@ced.uab.es) ) or Aïda Solé-Auró (aida.sole@upf.edu). Those wishing to request access to the health claims data of the AOK may contact WIdO (webpage: http://www.wido.de/, mail: wido@wido.bv.aok.de).

## Abbreviations

AOK: Allgemeine Ortskrankenkasse
CI: Confidence interval
CLARA: Clustering Large Applications
DEM: Dementia
DM: Diabetes mellitus
HEALIN: Health Inequality
HTN: Hypertension
ICD-10: International Statistical Classification of Diseases and Related Health Problems (10th edition)
M2Q: Minimum of two quarters
PAM: Partitioning Around Medoids
SVR: Subsequence vector representation
