# Supplementary material for: Examining morbidity and mortality trajectory profiles of hypertension, diabetes and dementia across healthcare systems: an analysis of Catalan and German administrative medical data for the years 2010 to 2019
Source: Popul Health Metr. 2026 Mar 29;24:26. doi: 10.1186/s12963-026-00473-6 (PMC13064399; doi:10.1186/s12963-026-00473-6)
Supplement: Supplementary file 1 — Supplementary Material 1. [file 12963_2026_473_MOESM1_ESM.docx]

**Additional information**

Table A1: Distribution of AOK population (age 50+) vs. total population by federal states

|  | **AOK, age 50+, first quarter 2004** | | **Total population 2004 at the beginning of the year** | | **Difference** |
| --- | --- | --- | --- | --- | --- |
|  | **n** | **%** | **n** | **%** | **percentage points (pp)** |
| ***Missing/abroad*** | 2680 | 1.1 | - | - | - |
| **Berlin** | 8872 | 3.6 | 3388 | 4.1 | -0.6 |
| **Baden-Württemberg** | 34347 | 13.8 | 10693 | 13.0 | 0.8 |
| **Bavaria** | 39656 | 15.9 | 12423 | 15.1 | 0.8 |
| **Bremen** | 1948 | 0.8 | 663 | 0.8 | 0.0 |
| **Hesse** | 16037 | 6.4 | 6089 | 7.4 | -1.0 |
| **Hamburg** | 2894 | 1.2 | 1734 | 2.1 | -0.9 |
| **Lower Saxony** | 21795 | 8.7 | 7993 | 9.7 | -1.0 |
| **North Rhine-Westphalia** | 39760 | 15.9 | 18080 | 21.9 | -6.0 |
| **Rhineland-Palatinate** | 11616 | 4.7 | 4059 | 4.9 | -0.3 |
| **Schlesweig-Holstein** | 7576 | 3.0 | 2823 | 3.4 | -0.4 |
| **Saarland** | 3153 | 1.3 | 1061 | 1.3 | 0.0 |
| **Brandenburg** | 9951 | 4.0 | 2575 | 3.1 | 0.9 |
| **Mecklenburg-Western Pomerania** | 6707 | 2.7 | 1732 | 2.1 | 0.6 |
| **Saxony** | 21761 | 8.7 | 4321 | 5.2 | 3.5 |
| **Saxony-Anhalt** | 10534 | 4.2 | 2523 | 3.1 | 1.2 |
| **Thuringia** | 10411 | 4.2 | 2373 | 2.9 | 1.3 |
| **Total** | 249698 | 100.0 | 82530 | 100.0 |  |

Source: AOK 2004-2019, [32].

Figure A1a: Catalan Cluster Quality Indices for 2 to 12 Groups.

**
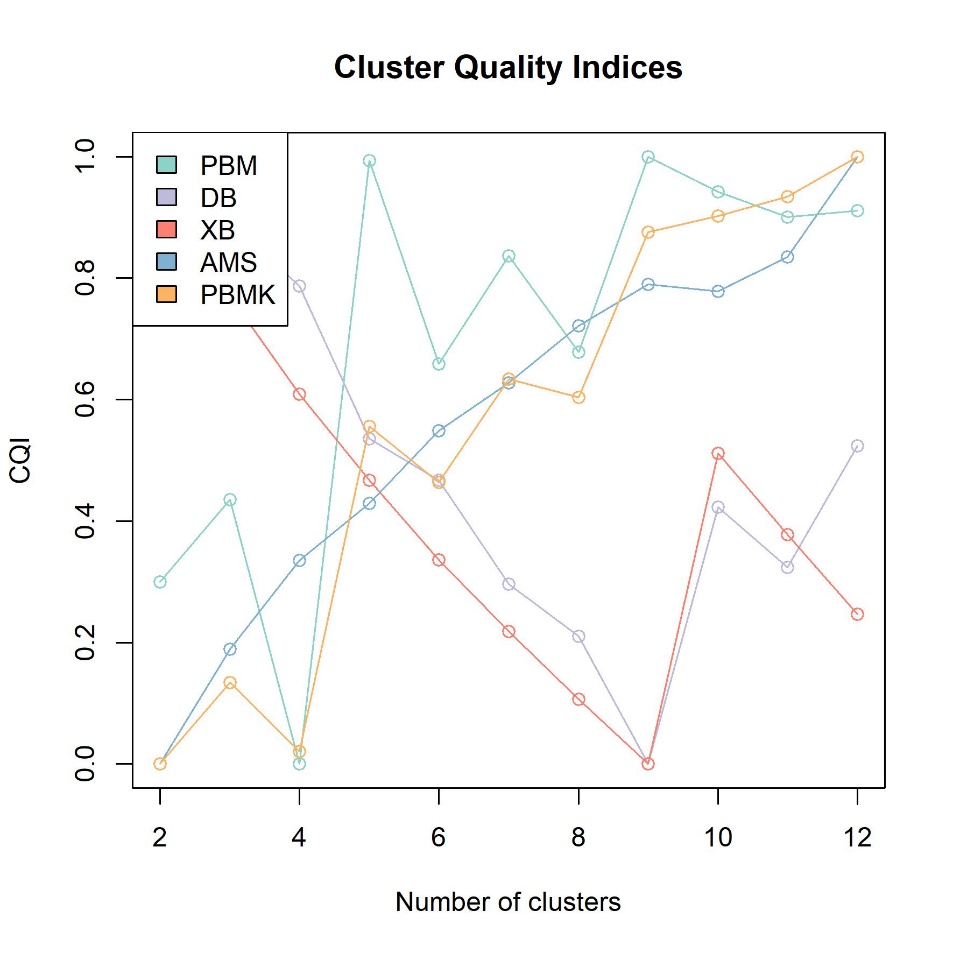
**

Legend: PBM – PBM Index (optimal index value: maximum); DB – Davies-Bouldin Index (optimal index value: minimum); XB – Xie-Beni Index (optimal index value: minimum); AMS – Average medoid silhouette with (optimal index value: maximum); PBMK – PBM Index less penalized by complexity (optimal index value: maximum). For further details, see [39]. Source: HEALIN (2005-2021).

Figure A1b: German Cluster Quality Indices for 2 to 12 Groups.


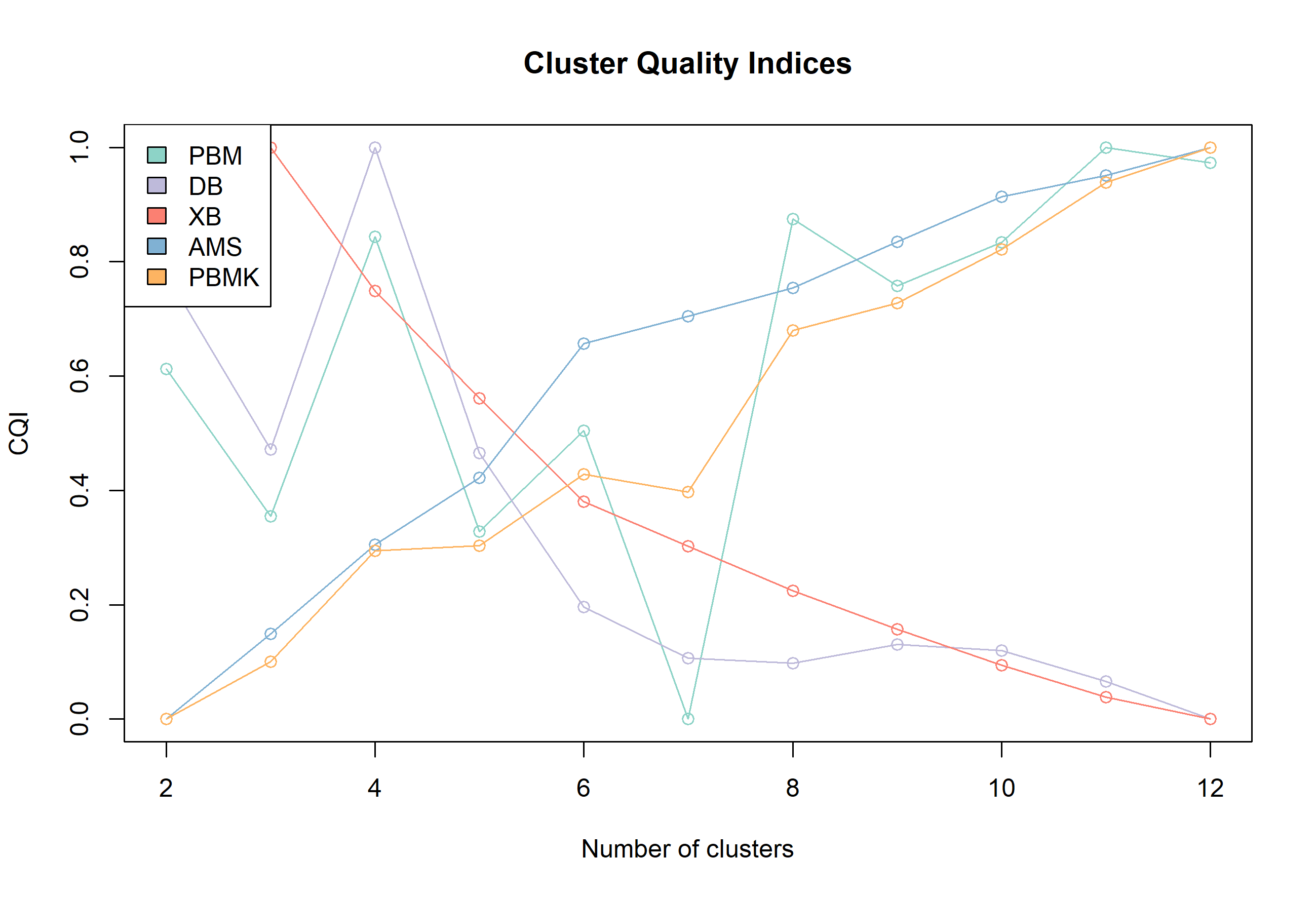


Legend: PBM – PBM Index (optimal index value: maximum); DB – Davies-Bouldin Index (optimal index value: minimum); XB – Xie-Beni Index (optimal index value: minimum); AMS – Average medoid silhouette with (optimal index value: maximum); PBMK – PBM Index less penalized by complexity (optimal index value: maximum). For further details, see [39]. Source: AOK (2004-2019).

Figure A2a: Mean Catalan clustering stability among CLARA iterations. Adjusted Rand Index (ARI) and Jaccard Coefficient (JC), also for 20% best performing iterations.
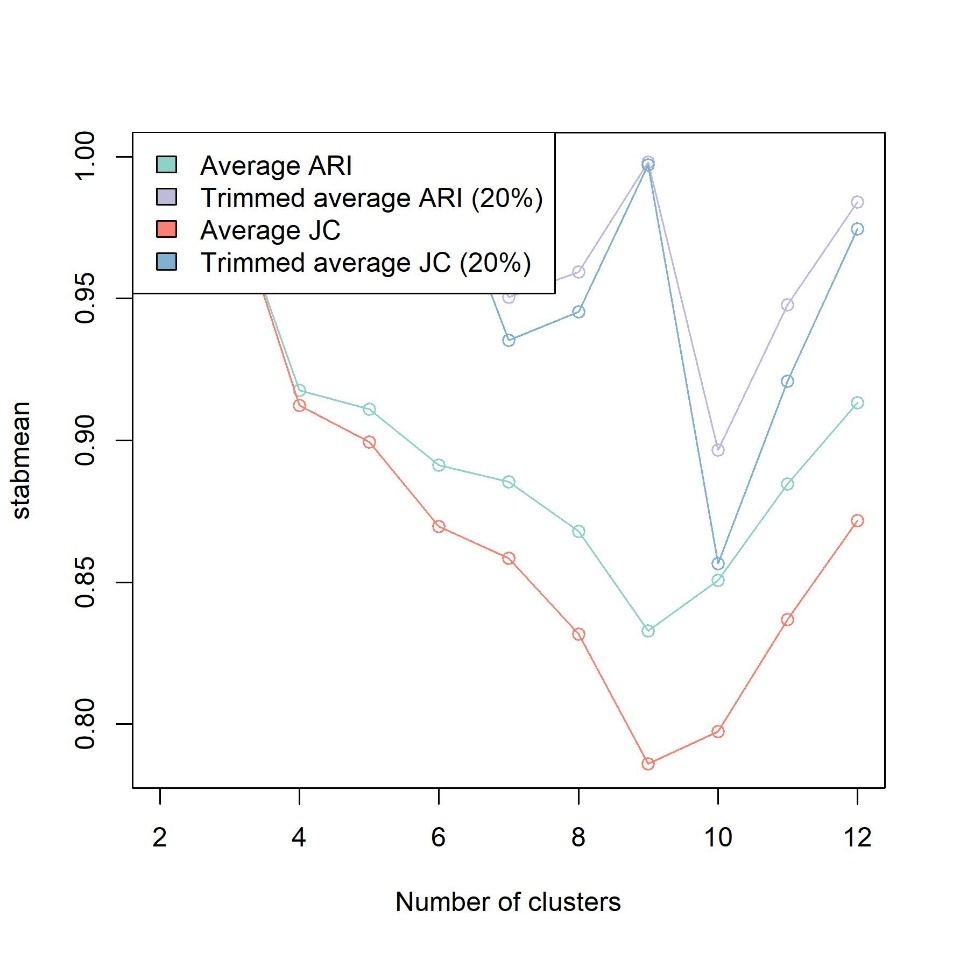


Legend: ARI – Adjusted Rand Index; CJ – Jaccard Coefficient; Trimmed average – the average among the best 20% of the iterations. Optimal stability values: maximum. For further details, see [39]. Source: HEALIN (2005-2021).

Figure A2b: Mean German clustering stability among CLARA iterations. Adjusted Rand Index (ARI) and Jaccard Coefficient (JC), also for 20% best performing iterations.
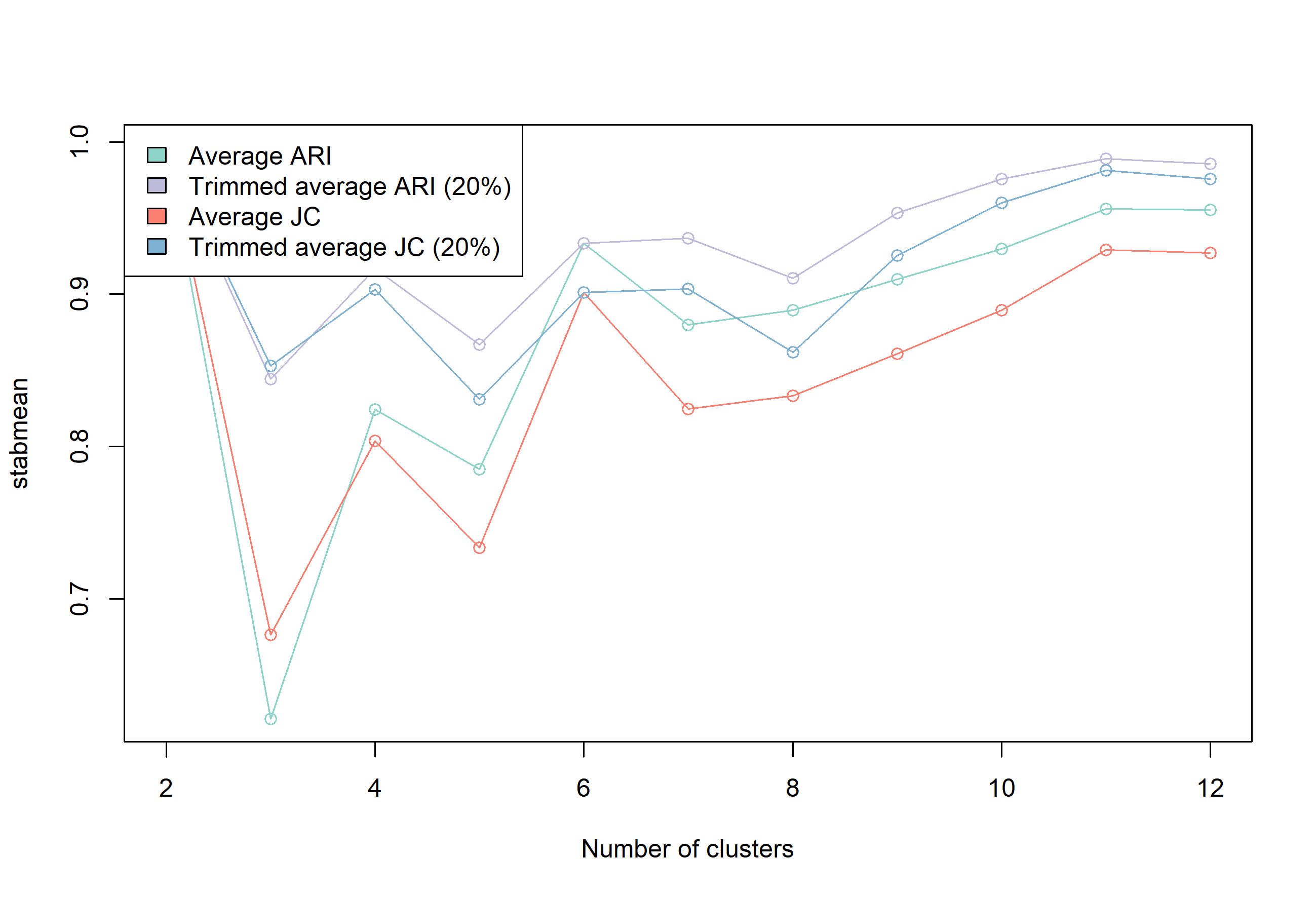


Legend: ARI – Adjusted Rand Index; CJ – Jaccard Coefficient; Trimmed average – the average among the best 20% of the iterations. Optimal stability values: maximum. For further details, see [39]. Source: AOK (2004-2019).

Table A2a: Relative risk to be in trajectory profile by birth cohort and sex, Catalan population, n = 174,798.

| **Relative risk** | **No disease I* (Ref.)** | **No disease II** | **No disease III** | **No disease IV** | **No disease V** | **No disease VI** | **No disease VII** | **Hypertension I*** | **Hypertension II*** | **Hypertension IV*** | **Diabetes / Diabetes + Hypertension I** |
| --- | --- | --- | --- | --- | --- | --- | --- | --- | --- | --- | --- |
| **(95% CI)** |  |  |  |  |  |  |  |  |  |  |  |
|  |  |  |  |  |  |  |  |  |  |  |  |
| **Intercept** | 1.00 | 0.31 | 0.16 | 0.09 | 0.04 | 0.06 | 0.01 | 0.73 | 0.11 | 0.08 | 0.56 |
|  |  | (0.30-0.32) | (0.15-0.17) | (0.08-0.09) | (0.04-0.05) | (0.05-0.06) | (0.01-0.01) | (0.70-0.75) | (0.10-0.12) | (0.08-0.09) | (0.54-0.58) |
| **Sex (Ref. Male)** | 1.00 | 1.00 | 1.00 | 1.00 | 1.00 | 1.00 | 1.00 | 1.00 | 1.00 | 1.00 | 1.00 |
|  |  |  |  |  |  |  |  |  |  |  |  |
| Female | 1.00 | 0.60 | 0.67 | 0.57 | 1.16 | 0.44 | 0.61 | 0.93 | 0.67 | 0.40 | 0.55 |
|  |  | (0.57-0.62) | (0.64-0.71) | (0.53-0.61) | (1.10-1.22) | (0.41-0.46) | (0.57-0.66) | (0.91-0.96) | (0.63-0.72) | (0.38-0.42) | (0.54-0.57) |
| **Birth cohort (Ref. 1950-54)** | 1.00 | 1.00 | 1.00 | 1.00 | 1.00 | 1.00 | 1.00 | 1.00 | 1.00 | 1.00 | 1.00 |
| 1945-49 | 1.00 | 1.07 | 1.14 | 0.97 | 1.77 | 1.43 | 1.87 | 1.17 | 1.03 | 1.61 | 1.25 |
|  |  | (1.01-1.13) | (1.07-1.23) | (0.88-1.07) | (1.60-1.97) | (1.28-1.61) | (1.45-2.41) | (1.13-1.22) | (0.94-1.12) | (1.46-1.77) | (1.20-1.31) |
| 1940-44 | 1.00 | 1.25 | 1.36 | 1.11 | 3.69 | 2.44 | 5.92 | 1.46 | 1.00 | 3.24 | 1.73 |
|  |  | (1.18-1.32) | (1.26-1.46) | (1.00-1.24) | (3.35-4.07) | (2.19-2.73) | (4.72-7.43) | (1.40-1.52) | (0.91-1.10) | (2.96-3.55) | (1.65-1.81) |
| 1935-39 | 1.00 | 1.38 | 1.35 | 1.04 | 6.70 | 5.02 | 21.35 | 1.63 | 0.97 | 6.74 | 2.37 |
|  |  | (1.29-1.47) | (1.24-1.46) | (0.92-1.18) | (6.09-7.37) | (4.51-5.59) | (17.22-26.46) | (1.56-1.71) | (0.87-1.07) | (6.17-7.37) | (2.26-2.48) |
| 1930-34 | 1.00 | 1.67 | 1.29 | 1.16 | 10.52 | 10.85 | 62.48 | 1.82 | 0.80 | 16.14 | 3.87 |
|  |  | (1.56-1.78) | (1.18-1.41) | (1.02-1.32) | (9.57-11.56) | (9.79-12.02) | (50.62-77.11) | (1.74-1.91) | (0.70-0.90) | (14.81-17.59) | (3.69-4.07) |

Legend: * profile also in German population; 95% confidence interval (CI) in parenthesis. Source: HEALIN Cohort 2005-2019.

Table A2b: Relative risk to be in trajectory profile by birth cohort and sex, German population, n = 121,547.

| **Relative risk** | **No disease I* (Ref.)** | **Hypertension I*** | **Hypertension II*** | **Hypertension III** | **Hypertension IV*** | **Hypertension V** | **Diabetes I** | **Hypertension & Diabetes I** | **Hypertension & Diabetes II** | **Hypertension & Diabetes III** | **Hypertension & Diabetes IV** |
| --- | --- | --- | --- | --- | --- | --- | --- | --- | --- | --- | --- |
| **(95% CI)** |  |  |  |  |  |  |  |  |  |  |  |
|  |  |  |  |  |  |  |  |  |  |  |  |
| **Intercept** | 1.00 | 1.09 | 0.36 | 0.04 | 0.22 | 0.02 | 0.30 | 0.61 | 0.03 | 0.16 | 0.02 |
|  |  | (1.04-1.13) | (0.34-0.38) | (0.04-0.05) | (0.21-0.24) | (0.02-0.03) | (0.28-0.32) | (0.58-0.64) | (0.03-0.04) | (0.15-0.18) | (0.02-0.03) |
| **Sex (Ref. male)** | 1.00 | 1.00 | 1.00 | 1.00 | 1.00 | 1.00 | 1.00 | 1.00 | 1.00 | 1.00 | 1.00 |
|  |  |  |  |  |  |  |  |  |  |  |  |
| Female | 1.00 | 1.35 | 1.15 | 1.25 | 0.59 | 0.70 | 0.66 | 0.99 | 1.08 | 0.54 | 0.70 |
|  |  | (1.30-1.40) | (1.09-1.21) | (1.17-1.34) | (0.56-0.62) | (0.65-0.75) | (0.62-0.70) | (0.95-1.03) | (1.01-1.16) | (0.51-0.56) | (0.66-0.75) |
| **Birth cohort (Ref. 1950-54)** | 1.00 | 1.00 | 1.00 | 1.00 | 1.00 | 1.00 | 1.00 | 1.00 | 1.00 | 1.00 | 1.00 |
| 1945-49 | 1.00 | 1.27 | 1.34 | 2.09 | 1.93 | 2.43 | 1.31 | 1.49 | 2.35 | 2.01 | 3.32 |
|  |  | (1.21-1.35) | (1.24-1.44) | (1.76-2.47) | (1.76-2.13) | (1.89-3.13) | (1.20-1.43) | (1.40-1.59) | (1.94-2.84) | (1.80-2.24) | (2.59-4.26) |
| 1940-44 | 1.00 | 1.44 | 1.48 | 5.04 | 3.10 | 7.71 | 1.25 | 1.84 | 5.27 | 3.64 | 10.38 |
|  |  | (1.36-1.52) | (1.37-1.59) | (4.35-5.83) | (2.84-3.38) | (6.21-9.58) | (1.14-1.37) | (1.73-1.95) | (4.45-6.23) | (3.29-4.02) | (8.33-12.94) |
| 1935-39 | 1.00 | 1.63 | 1.76 | 10.22 | 5.43 | 21.46 | 1.37 | 2.17 | 11.18 | 6.86 | 30.69 |
|  |  | (1.54-1.72) | (1.63-1.89) | (8.88-11.76) | (4.99-5.91) | (17.44-26.41) | (1.25-1.50) | (2.04-2.31) | (9.52-13.12) | (6.24-7.55) | (24.81-37.96) |
| 1930-34 | 1.00 | 1.60 | 1.62 | 17.35 | 11.46 | 70.33 | 1.60 | 2.09 | 17.59 | 13.45 | 88.00 |
|  |  | (1.50-1.71) | (1.48-1.77) | (15.02-20.04) | (10.48-12.53) | (57.18-86.51) | (1.44-1.78) | (1.94-2.25) | (14.92-20.74) | (12.17-14.87) | (71.09-108.93) |

Legend: * profile also in Catalan population; 95% confidence interval (CI) in parenthesis. Source: AOK 2004-2019.
